# Does the MUNIX Method Reflect Clinical Dysfunction in Amyotrophic Lateral Sclerosis: A Practical Experience

**DOI:** 10.1097/MD.0000000000003647

**Published:** 2016-05-13

**Authors:** Malgorzata Gawel, Magdalena Kuzma-Kozakiewicz

**Affiliations:** From the Department of Neurology (MG, MK-K); and Neurodegenerative Disease Research Group (MG, MK-K), Medical University of Warsaw, Poland.

## Abstract

The aim of our study was to assess the usefulness of the MUNIX method in reflecting the clinical dysfunction in patients with amyotrophic lateral sclerosis (ALS), as well as to assess an intra-rater reproducibility of MUNIX. The study group consisted of a total of 15 ALS patients. The mean age of symptoms onset was 55 years, and the mean disease duration was 10 months. The muscle strength and patients’ functional status were assessed according to the Medical Research Council (MRC) and by ALS functional rating scale revised (ALSFRS-R), respectively. The MUNIX was performed in 6 muscles: abductor pollicis brevis (APB), abductor digiti minimi (ADM), biceps brachii (BB), tibial anterior (TA), extensor digitorum brevis (EDB), and abductor hallucis (AH), unilaterally, at a less affected side. Both muscle-specific and global MRC and MUNIX scores were calculated. In 11 patients, the study protocol was repeated at least twice every 3 months. An additional testing of the intra-rater reliability was performed at the first visit.

There were no significant differences between MUNIX test and re-test values in the APB, ADM, BB, TA, EDB, and AH muscles (*P* >0.05). The highest variability of the test–retest values was found in the BB muscle (7.53%). Although there was a significant test–retest difference in the global MUNIX score (*P* = 0.02), the variability of the results was as low as 1.26%. The MUNIX value correlated with the muscle-specific MRC score in ABP, ADM, TA, EDB and AH (*P* <0.05), and the global MUNIX values correlated with global MRC scores (*P *<0.05). There was also a significant correlation between the global MUNIX score and the clinical dysfunction measured by the ALSFRS-R scale (*P* <0.05). The global MUNIX showed a higher monthly decline (4.3%) as compared with ALFRS-R (0.7%) and the MRC global score (0.5%).

This study confirms that the MUNIX method is a sensitive, reliable, and accurate tool reflecting both motor dysfunction and disease progression in ALS. We have found this approach to be more reliable and technically easier in distal muscles with less atrophy and a better strength.

## INTRODUCTION

Amyotrophic lateral sclerosis (ALS) is an adult-onset, progressive lethal neurodegenerative disorder characterized by a selective dysfunction and loss of upper and lower motor neurons. Within a few years from the first symptoms onset it leads to quadriplegia and respiratory insufficiency. ALS shows a characteristic variability of onset and a rate of disease progression, which together with a clinical heterogeneity makes the quantification of the symptoms problematic.^1^ It is therefore important to develop strategies that would allow to objectively assess the disease progression and predict the outcome.^2^ The diagnosis of ALS is based on a clinical evaluation together with conventional electromyography (EMG).^3^ Routine quantitative EMG is used for estimation of the presence of the lower motor neuron involvement and its consequences, including primary denervation and compensatory reinnervation of muscle fibers. It is not however useful in the assessment of a motor unit number reserve. Abnormal needle EMG recording reflects the effects of two overlapping processes that occur in muscles: acute denervation and reinnervation. In the first stage of the disease, the loss of anterior horn cells results in an acute motor fiber denervation. Afterwards, in the stage of a secondary innervation of muscle fibers, the denervation is compensated by sprouting of axonal collaterals from surviving motor units into denervated muscle fibers. Motor unit activity potential (MUAP) parameters are increased at the stage of secondary reinnervation. It is because of an enlarged motor unit area and a dispersion, which results from differences in the duration of potential components caused by abnormal neuromuscular transmission in immature axonal collaterals. Finally, in the stage of decompensation, the motor unit activity potential parameters decrease because of the continuous loss of motor units and a decrease of their area.^4^ Neurophysiological changes in motor units in ALS are in continuous evolution along with a dynamic reorganization.

Because of these overlapping processes, the motor unit activity potential parameters appear not to correlate with clinical muscle dysfunction. Pseudo-normal MUAP parameters may be observed even in the terminal stage of the disease as the MUAP parameters do not asses the motor unit number but the effect of denervation–reinnervation processes.

One of recently developed electrophysiological approaches, which may provide information about the degree of motor unit loss, is the motor unit number index (MUNIX) method.^5^ MUNIX is a noninvasive method that can be applied to any muscle in which a compound muscle action potential (CMAP) can be evoked by supramaximal nerve stimulation. The result of the examination is directly related to the number of functioning motor neurons in a given muscle. MUNIX uses a mathematical model based on the CMAP and the surface interference pattern following their import into analysis software created by Nandedkar et al.^5,6^ The result is presented as a plot and a numeric value reflecting the number and size of motor units recruited at various force levels. The aim of this study was to assess the usefulness of the MUNIX method in reflecting the clinical dysfunction in patients with ALS, as well as to assess reproducibility of the method. It was also important to understand the relationships between MUAP parameters and the clinical muscle dysfunction in patients with ALS in order to further use MUNIX as an electrophysiological marker of the muscle function in diseases with progressive motor unit loss.^7^

## MATERIAL AND METHODS

The study group included 15 patients with possible (n = 2), probable laboratory-supported (n = 1), and probable (n = 12) ALS according to the revised El Escorial criteria.^3^ The diagnosis was made by an experienced ALS specialists. The mean age of the disease onset was 55 years (range 28–75 years), and the mean duration of the symptoms (defined as the onset of weakness, muscle wasting, fasciculations, dysarthria, dysphagia, dyspnea, falls, or disturbance of fine finger movements) was 10 months. The symptom onset was localized in the arm region in 7 patients, in the leg region in 3 patients, in the bulbar region in 5 patients. After the recruitment visit, the patients were followed up for 15 months (whenever possible), in 3-month intervals.

The control group consisted of 12 healthy volunteers: 6 women and 6 men (mean age 48.6 years). Individuals with any history of major neurological disorders that might influence MUNIX measurements (e.g., polyneuropathy, peripheral nerve injury, and muscular disease) were excluded from the study.

We performed MUNIX measurements in the abductor pollicis brevis (APB), abductor digiti minimi (ADM), biceps brachii (BB), tibial anterior (TA), extensor digitorum brevis (EDB), and abductor hallucis (AH) muscles. MUNIX was recorded on the clinically less affected side of the body and all the measurements were taken on the same side. We used the Keypoint Classic Natus Apparatus. In case the CMAP amplitude was <0.5 mV, a given muscle was not used for MUNIX calculation and MUNIX was rated as zero.^5,20^

Before every MUNIX measurement, a manual muscle testing according to the expanded Medical Research Council scale for manual muscle testing (MRC) was performed in each investigated muscle, using the additional graduation M4– and M4+. The global MUNIX and MRC scores were calculated at each visit. In 11 patients, the study protocol was repeated at least twice (range 2–5 times) every 3 months. At the first visit, an additional testing of the intra-rater reliability was performed: the examination was repeated twice with an interval of minimum 30 minutes between sessions of test and retest. The ALS Functional Rating Scale (ALSFRS-R) was assessed at each visit.

The protocol was approved by the Bioethics Committee at the Medical University of Warsaw, and an informed consent was obtained from all of the patients.

All the raw data of this study was placed on the Progeny software: euromotor.umcutrecht.nl, an electronic platform created within the SOPHIA project “Sampling and biomarker Optimization and Harmonization in ALS and other motor neuron diseases” financed by EU Joint Programme—Neurodegenerative Disease Research (JPND, www.jpnd.eu, 2012–2017). The platform assures access to clinical and electrophysiological data of patients with ALS respecting the national data privacy policy.

Regression models were used to calculate generalized linear mixed models. As a probability distribution random factor is assumed: normal distribution when variability remained constant over time, and the gamma distribution when the variability of the test parameter increased over time. The repeatability of the measurements was examined by calculating the average of the difference between repetitions and estimating what proportion of the total variation is the variability associated with repeated measurements (internal correlation). Preliminary correlations were assessed using the Pearson and Spearman's coefficients.

Correlations to account for the effect of “clustered data” (data with a hierarchical structure) and the initial symptom “factor” have been counted on the basis of the determination coefficient calculated using the generalized linear mixed models. Trend analysis for the duration of the disease for the first visit and also for all visits was carried out using regression models that incorporated generalized linear mixed models internal correlations and “confounding factors.” Statistical analysis was performed using SAS software, version 13.2. *P* <0.05 was considered statistically significant. Mean monthly percentage reductions of the MUNIX value compared with the baseline value in all examined muscles and of the ALS Functional Rating Scale score during the follow-up were calculated.

## RESULTS

The mean global MUNIX in ALS patients was significantly lower than in healthy volunteers. A significant difference between MUNIX values in healthy controls and ALS patients was found in APB, ADM, AH, extensor digitorum but not in BB and TA (Table 1).

**TABLE 1:**
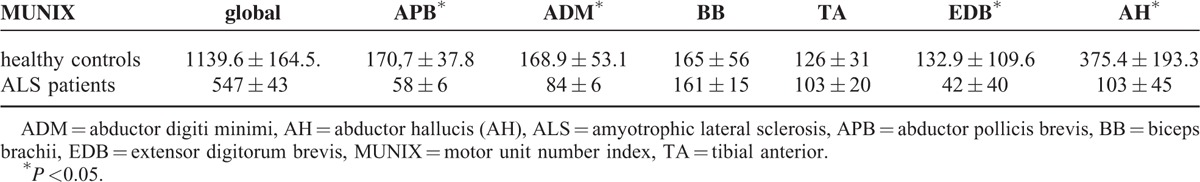
MUNIX Values in ALS Patients and Controls

There was no significant difference between MUNIX test and re-test values in the APB, ADM, BB, TA, EDB, and TA muscles (*P* >0.05). The most marked variability between test and re-test values was found in the BB muscle (7.53%). Although there was a significant test–retest difference in the global MUNIX score (*P* = 0.02), the variability of the results was low (1.26%).

**FIGURE 1:**
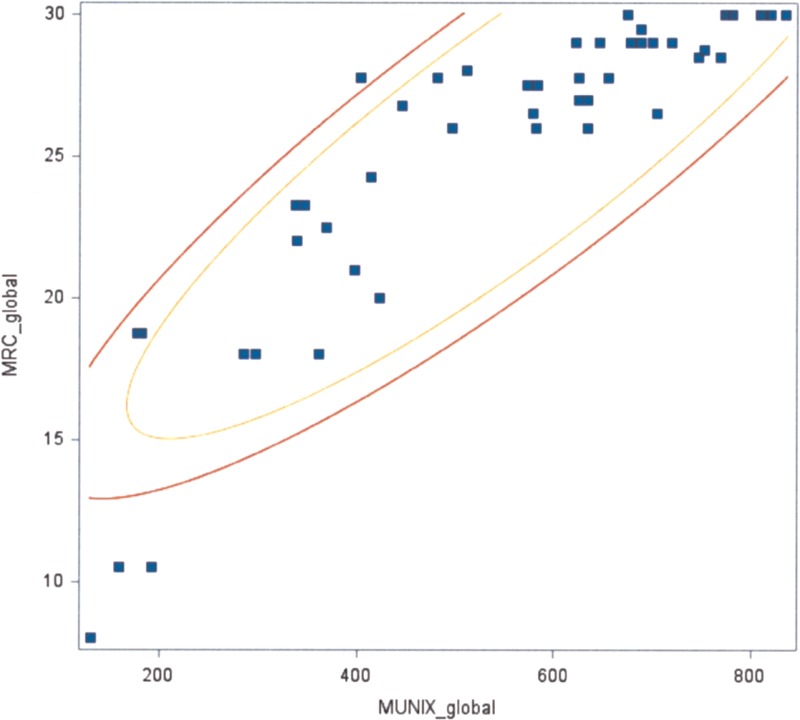
Correlation between the global MUNIX score and the global MRC scale (*P* <0.05). MRC = Medical Research Council, MUNIX = motor unit number index.

**FIGURE 2:**
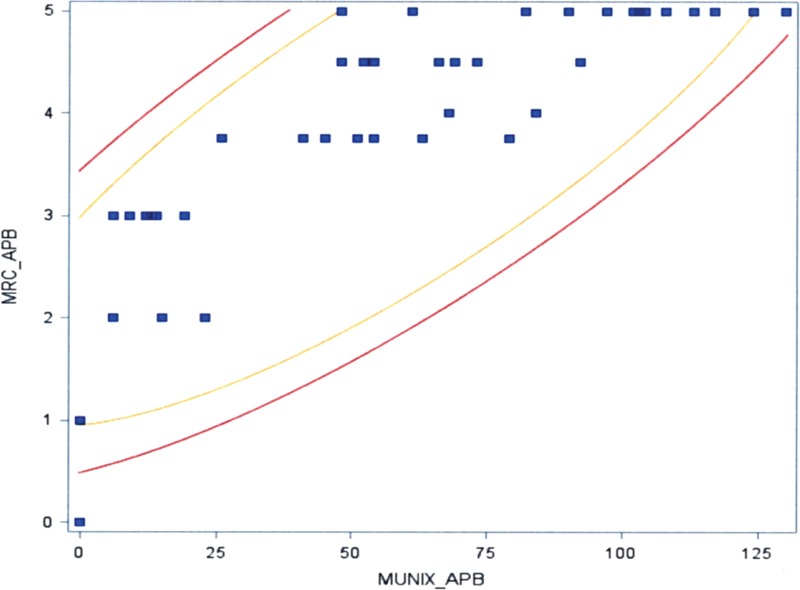
Correlation between MUNIX in the APB and the MRC score (*P* <0.05). APB = abductor pollicis brevis, MRC = Medical Research Council, MUNIX = motor unit number index.

**FIGURE 3:**
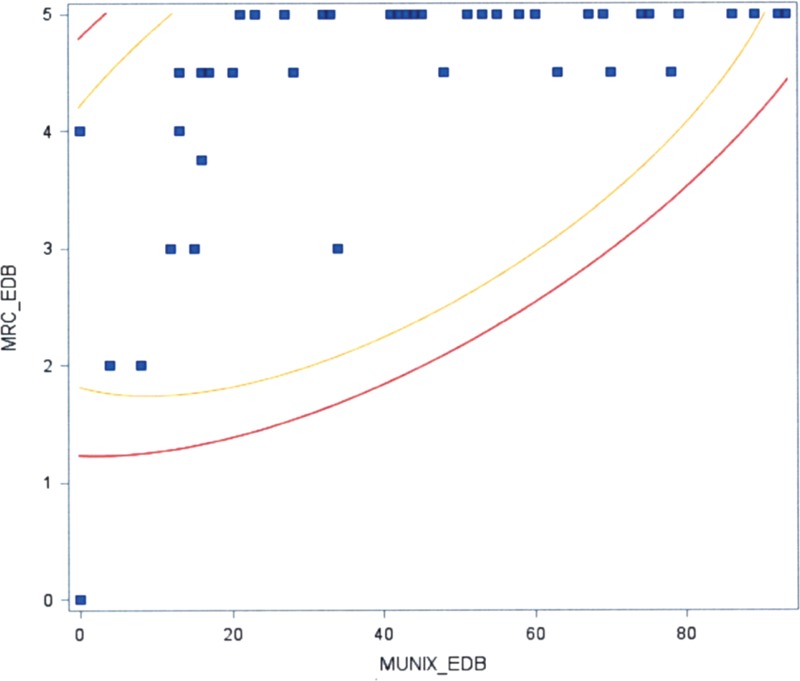
Correlation between MUNIX in the EDB and the MRC score (*P* <0.05). EDB = extensor digitorum brevis, MRC = Medical Research Council, MUNIX = motor unit number index.

**FIGURE 4:**
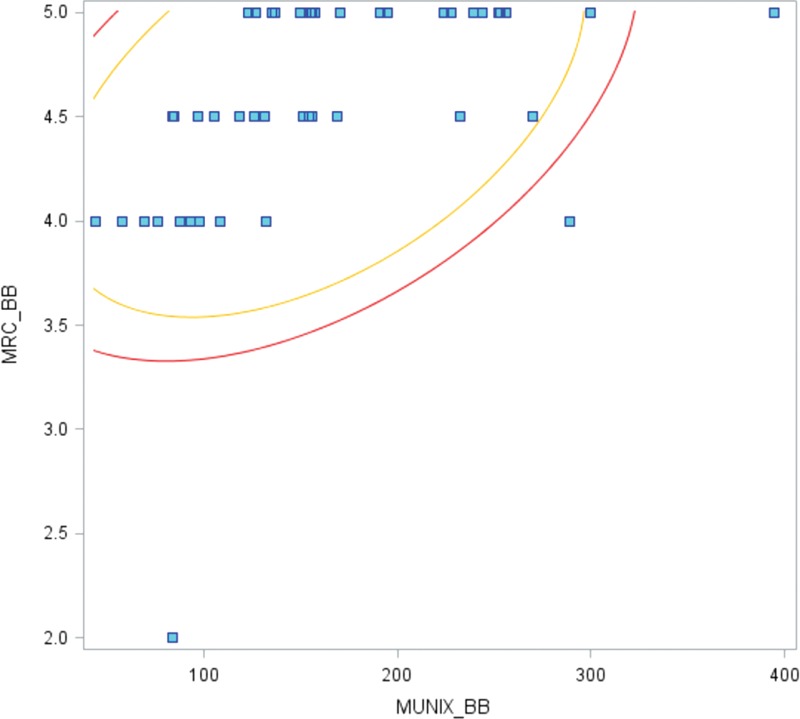
Correlation between MUNIX in the BB and the MRC score (*P* <0.1). MRC = Medical Research Council, MUNIX = motor unit number index.

The global MUNIX values correlated with global MRC scores (*P* <0.05) (Figure 1). The MUNIX value correlated with the MRC score for each of the following muscles: APB (*P* <0.05), ADM (*P* <0.05), TA (*P* <0.05), EDB (*P* <0.05), and AH (*P* <0.05) (Figures 2 and 3). A similar trend was also found in the BB (*P* <0.1) (Figure 4).

**FIGURE 5:**
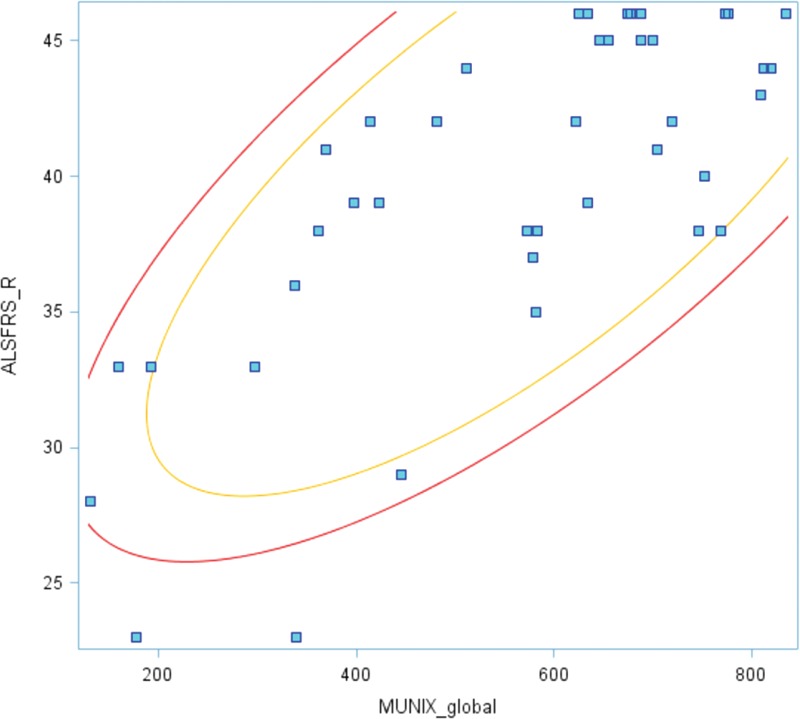
Correlation between the global MUNIX score and the ALSFRS-R scale (*P* <0.05). ALSFRS-R = amyotrophic lateral sclerosis functional rating scale revised, MUNIX = motor unit number index.

There was a significant correlation between the global MUNIX score and the ALS Functional Rating Scale (*P* <0.05) (Figure 5) but not between MUNIX and the disease duration.

Additionally, in 12 patients we were able to analyze the relationship between MRC and motor unit activity potential parameters (amplitude, duration, and size index) obtained in the BB muscle during a slight voluntary effort.^8–10^ The mean MUAP amplitude was 827.6 ± 336.6 mV, mean MUAP duration was 10.76 ± 1.3 ms, and mean MUAP size index was 1.22 ± 0.56. The mean MRC value in the BB muscle was 4.62 ± 0.43. No correlation was found between MUAP parameters and MRC values in the BB muscle. The Spearman coefficient was calculated retrospectively for the amplitude (*r* = −0.28, *P* = 0.37), the duration (*r* = −0.32, *P* = 0.31), and the size index (*r* = −0.43, *P* = 0.16).

**FIGURE 6:**
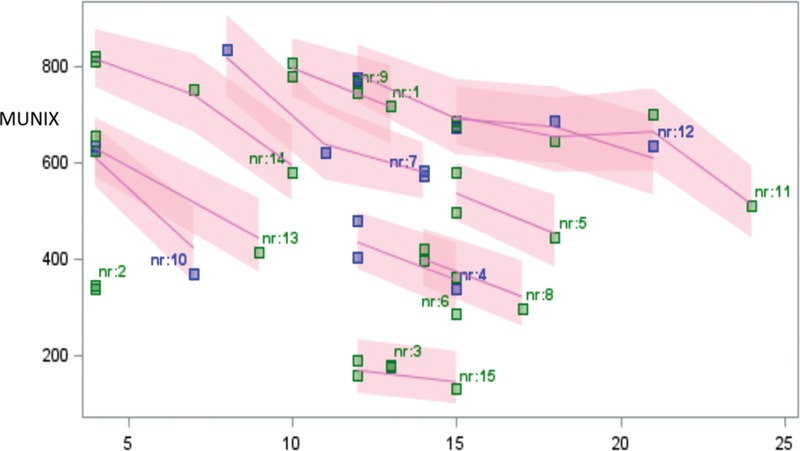
Trends for the MUNIX global score decline in ALS patients at subsequent assessments. ALS = amyotrophic lateral sclerosis, MUNIX = motor unit number index.

ALS functional rating scale declined from the first to the last visit by 0.7% per month. The mean monthly relative decline was 1.6% in ADM, 5.6% in APB, 4.02% in BB, 1.7% in TA, 3.7% in EDB, and 1.5% in AH. The most significant decline was observed in the APB muscle. The mean monthly decline of the global MUNIX score was 4.3% (Figure 6) and this downward trend was more marked compared with the monthly decline in the ALS functional rating scale (0.7%) or the MRC global score (0.5%).

## DISCUSSION

In addition to clinical methods used for monitoring the disease progression, the quantitative methods more directly related to the underlying disease pathogenesis are of great interest. If efficient, they may become new outcome measurements in therapeutic trials.^11^ Our experience with motor unit number estimation (MUNE) methods started with MUNE in the Shefner's modification.^12,13^ The method was used to measure the progress of motor neuron loss in physiological aging^14^ and in diseases with motor neuron degeneration in the anterior horns of the spinal cord, as well as in spinal muscular atrophy and ALS.^15,16^ Our studies revealed that the MUNE method was a noninvasive, easy to perform approach, and with a very low variability. We were able to prove that it could be a useful tool not only for estimation of disease progression but also as a complementary method for the initial muscle assessment. However, the most significant limitation of this method is its usefulness only in the distal muscles, what makes it of little use in the generalized stage of the disease. In contrast, the MUNIX method can be used in both distal and proximal muscles. Moreover, MUNIX only depends on the maximal compound motor activity potential measurement and the recording of surface EMG interference patterns at different grades of voluntary muscle contraction. It does not require repeated stimulation to obtain a compound motor activity potential. With this, the method circumvents the problem occurring with the incremental MUNE method when several axons with similar stimulation thresholds may fire at the given stimulus intensity, leading to variable amplitude (“alteration”) during repetitive stimuli.^11,17^

Multiple studies have tested the MUNIX method in a single, more frequently a distal muscle. On the contrary, our study is one of a few approaches using the MUNIX method in multiple muscles in ALS patients, able to demonstrate that the loss of lower motor neurons can be tracked. As it was shown previously, changes in the motor unit number estimated by MUNE or MUNIX appear to have a greater sensitivity for disease progression compared with other clinical biomarkers such as compound motor activity potential amplitude, grip strength, MRC grade of weakness, and forced vital capacity,^18^ and the changes are even seen before the clinical onset of the disease.^19^ Our results are comparable with other prospective studies of disease progression in ALS, revealing that MUNE changes are more sensitive than other measures of lower motor neuron dysfunction.^20^

The present study (with intra-rater test–retest) revealed that MUNIX is reproducible in patients with ALS. Similarly to the results of Neuwirth et al, our analysis revealed that the highest test–retest MUNIX variability was found in the BB muscle. It can be explained by technical difficulties with obtaining maximal compound motor activity potential amplitude because of suboptimal electrode position, probably related to the fact that BB has the biggest mass among all examined muscles. Moreover, the BB and TA muscles were excluded from some analyses of Neuwirth et al because the stimulation of the musculocutaneus nerve was technically challenging and a costimulation of neighboring nerves could result in erroneously high compound motor activity potential amplitudes.^21^ Further studies are needed to improve the reproducibility of BB testing. It should also be noted that an increased variability was found in weaker muscles with markedly reduced MUNIX values.

Similarly to other authors, we have found the decline of MUNIX in several muscles to be significantly steeper than the functional decline as reflected by ALSFRS-R. In contrast to other studies,^21^ the most prominent percentage changes per month were seen in the small hand muscles (APB) but also in the BB.

The conventional EMG reveals the consequences of the overlapping processes caused by degeneration and reorganization of motor units. This reorganization of the motor unit resulting from coexisting processes of denervation and reinnervation, initially allows a full compensation, but it finally leads to a decompensation of muscle fiber innervation. In the initial disease stage, the values of MUAP parameters are increased because of efficient reinnervation of muscle fibers by collateral axonal sprouting (for the amplitude of ∼500% normal value, for duration ∼40% normal value).^21^ In the end stage of the disease with advanced muscle atrophy, the MUAP parameters could be normal because of a decompensation of re-innervation and disintegration of the motor unit. In our study, we found no correlation between MUAP parameters and the MRC score in the BB, which confirms that in contrast to MUNIX, the MUAP parameters do not reflect the clinical muscle dysfunction. On the contrary to the routine EMG, MUNIX is a good indicator of muscle clinical dysfunction.

During the study, we made some practical observations. The MUNIX method may be affected by an improper compound motor activity potential recording caused by technical or anatomical problems. It is crucial to find the optimal location of the recording electrode and the repeat compound motor activity potential recording to make sure that the motor response with maximal amplitude is obtained. However, recurrent stimuli may result in a habituation and decreased potential amplitude; therefore, the intervals between stimuli are required. Lower compound motor activity potential amplitude has a dramatic effect on the reduction of MUNIX. When a potential is recorded with a stimulus artifact, the power cannot be measured accurately and nor can the MUNIX. Because of the need to perform a gradual effort, a good cooperation with the patient is crucial.

Our results suggest that MUNIX is a method that may be useful not only for estimation of disease progression but also as a complementary method for initial assessment of muscle. From practical point of view the global MUNIX seems to be more useful in the general patient assessment. The MUNIX (global and muscle-specific) seems to be a good biomarker of disease progression to be used in clinical trials.^10,20^

## CONCLUSION

This study confirms that the MUNIX method is a sensitive tool reflecting motor dysfunction in ALS. Since MUNIX directly assesses the loss of the lower motor neurons, it may also be a good biomarker of ALS progression. However, we have found this approach to be more reliable and technically easier in distal muscles with less atrophy and better strength.
